# Dietary Intake and Energy Expenditure Assessed during a Pre-Season Period in Elite Gaelic Football Players

**DOI:** 10.3390/sports7030062

**Published:** 2019-03-13

**Authors:** Luke O’Brien, Kieran Collins, Dominic Doran, Omid Khaiyat, Farzad Amirabdollahian

**Affiliations:** 1 School of Health Sciences, Liverpool Hope University, Hope Park, Liverpool L16 9JD, UK; alizado@hope.ac.uk (O.K.); amirabf@hope.ac.uk (F.A.); 2 Gaelic Sports Research Center, Institute of Technology Tallaght, Tallaght, 24 Dublin, Ireland; kieran.collins@it-tallaght.ie; 3 The Tom Reilly Building, Research Institute for Sport and Exercise Sciences, Liverpool John Moores University, Liverpool L3 5UA, UK; d.a.doran@ljmu.ac.uk

**Keywords:** Gaelic sport, dietary intake, energy expenditure, macronutrients, training load, pre-season

## Abstract

There is currently a lack of research into the energy demands and associated nutritional intakes of elite Gaelic football players during the pre-season period, which is a crucial time of year for physical development. The aim of the current study was to investigate the dietary intake and energy expenditure (EE) of elite Gaelic football players during a typical pre-season week. Over a seven-day period, which included four training days and three rest days, dietary intake (validated self-reported estimated food diary) and EE (Sensewear Pro armband) were recorded in 18 male players from a single elite inter-county Gaelic football team. Average energy intake (EI) (3283 ± 483 kcal) was significantly (*p* = 0.002) less than average EE (3743 ± 335 kcal), with a mean daily energy deficit of −460 ± 503 kcal. Training days elicited the greatest deficits between intake and expenditure. The mean carbohydrate (CHO) intake was 3.6 ± 0.7 g/kg/day, protein intake was 2.1 ± 0.5 g/kg/day, and fat intake was 1.6 ± 0.2 g/kg/day. These findings indicate that the dietary practices of the sampled players were inadequate to meet EE and CHO recommendations. Training days are of particular concern, with the players not altering energy and CHO intake to encounter increased energy demands. Education on nutritional strategies for elite Gaelic footballers should be considered in relation to training demands to avoid detriments to performance and health.

## 1. Introduction

Gaelic football is an intermittent field-based team sport characterised by irregular bouts of high-intensity efforts (e.g., sprinting, striding, tackling, jumping) interspersed by less intense activity (e.g., walking and jogging) [1]. Elite Gaelic football players have been shown to cover on average between 8160 ± 1482 m and 9222 ± 1588 m of total distance during match play with between 1596 ± 594 m and 1731 ± 659 m of this completed at high speed (≥17 km/h) [2,3,4]. In terms of relative distance, players cover 116 ± 22 m/min to 131 ± 22 m/min [2,3,4]. The relative distance that elite Gaelic footballer cover during match play are similar to elite Australian rules footballers (128 m/min) [5] and soccer players (122 m/min) [6], and higher than that of rugby league backs (109 m/min) [7]. It is imperative for players to develop and maintain physical fitness in order to cope with the match play demands of the game and reduce the risk of injury [8].

Gaelic footballers are amateur athletes; however, elite Gaelic footballers’ participate in high volume and periodised training comparable to professional team sport athletes [9]. Elite Gaelic footballer’s playing cycle can be divided into ‘off-season’, ‘pre-season’, and ‘in-season’. During the pre-season period, elite Gaelic footballers commonly train two to three times on the pitch per week, which is supplemented by one to two gym sessions, while attempting to balance a professional (work/study) life [9]. The pre-season is a crucial time of the year for physiological adaptation; its main aim is to condition the players so that they can cope with the physical demands of the game following a deconditioning period during the off-season [10]. An important objective for this period is developing player’s aerobic capacity, strength, power, and speed alongside optimising body composition [10,11].

Changes in the physiological and anthropometrical characteristics over the duration of a season have been identified in Gaelic football players. Kelly and Collins [11] reported that in an elite Gaelic football squad of 26 players, mean body mass increased across the season; however, the change was non-significant. In contrast, the sum of eight skinfolds and percentage of body fat (% BF) significantly decreased between pre-season and mid-season. The pre-season is usually the time at which aerobic capacity, speed, strength, and power development are incorporated into the periodisation of Gaelic football training [11]. Kelly and Collins [11] have reported performance improvements in sprint times over 5 m and 10 m, counter movement jump height, and the Yo-Yo Intermittent Recovery Test Level 2 (YYIRT2), from early pre-season to early in-season. Performance improvements are less obvious from early in-season to mid-season, indicating that the training is shifting towards a more technical focus with a reduction in direct fitness work [12].

Despite the crucial role that nutrition plays in promoting optimal performance, supporting training adaptations, and maintaining immune function [13], little attention has been given to the energy requirements and dietary intake of Gaelic footballers during the pre-season, which is a phase that is characterised by high-intensity training sessions [12]. To date, only two published studies have investigated the dietary intakes of elite Gaelic football players [14,15]; however, none have specifically examined the energy and macronutrient intakes during a pre-season period. Furthermore, there is no data evaluating nutritional intake alongside the energy expenditure (EE) of elite Gaelic footballers. Several studies have reported that athletes fail to compensate for the increased energy demands of training and competition; despite fluctuations in training load and EE, dietary intake remains consistent [16,17]. Information is lacking in the literature to determine if Gaelic football players are adjusting their dietary intake to account for changes in training load throughout the week.

It is well recognised that carbohydrates (CHOs) are an important fuel source for high-intensity aerobic team based sports such as Gaelic football [18]. The most recent guidelines for nutritional intake in team sports recommend a daily CHO intake between 5–7 g/kg/day on moderate intensity training days, and between 6–10 g/kg/day mass for heavy training days or for match preparation [13]. However, research has reported that athletes across a range of team sports consistently fail to meet CHO recommendations [16,19,20,21]. This trend is also present in the two studies that have analysed the dietary intake of elite Gaelic footballers [14,15]. Daily variations in training demands and energy requirements determine the need to adjust dietary CHO intake on a day-to-day basis [13]. In addition to sufficient CHO intake, the intake of adequate amounts of protein is vital to optimise recovery from and enhance adaptations to exercise [22]. The recommended total daily protein requirements for team sport athletes range between 1.2–2.0 g/kg/day [13]. Unlike CHO intake, the nutritional assessment of Gaelic footballers in previous studies revealed adequate protein intakes [14,15].

The nutritional intake of elite players should match the fuel requirements of training and support training adaptations [13]. Although there have been many studies on the energy intake (EI) and EE of other team sport athletes [16,19,20], there is no data evaluating the nutritional intake alongside the EE of elite Gaelic footballers. This data would allow Gaelic football nutritionists and strength and conditioning practitioners to make informed decisions with regard to player’s nutritional intake during the preparatory phase. Therefore, the aim of this study was to examine energy and macronutrient intakes alongside EE during a pre-season training week. Based on the available evidence obtained from elite Gaelic footballer players and other adult male team sport athletes, it was hypothesised that: EI would be significantly less than EE, the dietary CHO intake of players would fail to meet recommended guidelines, and players would not alter energy and CHO intake to encounter the increased energy demands of training.

## 2. Materials and Methods

### 2.1. Study Design

This study assessed the dietary intake and EE of elite Gaelic football players for the first time across a seven-day pre-season period, which include two pitch training days, two gym training days, and three rest days. Players wore SenseWear armbands and completed a detailed seven-day food diary to assess energy expenditure and dietary intake. A summary of the pre-season weekly training plan can be seen in Table 1.

### 2.2. Participants

Eighteen male Gaelic football players from an elite Gaelic football squad (mean ± SD; age 23.7 ± 3.1 years, body mass 84.6 ± 8.0 kg, height 1.83 ± 0.06 m) volunteered to participate in this study. All of the players were older than 18 years of age, and at the time of testing, were free from any known illness. All of the players were involved in regular training over the study duration, fully informed of the study procedures before giving their written informed consent to participate, and free to withdraw from the study at any time. The study received ethical approval from the institutional ethics committee (S 20-04-2017 PA 009).

### 2.3. Anthropometry

Body mass was determined to the nearest 0.1 kg using digital scales (Seca, Birmingham, UK) while the participants wore only shorts. Height was measured to the nearest 0.1 cm using a portable stadiometer (Seca, Birmingham, UK). The participants maintained an upright posture with their feet together and heels touching the base of the stadiometer.

### 2.4. Dietary Intake

Player’s energy and macronutrient intakes were assessed using a seven-day paper-based food diary (7dFD). This time period is considered to provide accurate estimations of dietary intake whilst achieving high compliance [23]. Players were instructed to log a complete record of all foods and drink consumed over a seven-day period. Specific training on completing the food diary was provided by the lead researcher who is a registered nutritionist (Association for Nutrition). Detailed written instructions for completing the food diary were also provided, and a sample page of an accurately completed food record was included. All of the players were instructed not to change their habitual diet during the recording period, and asked to be as accurate as possible in recording the time of food consumption, amount (household measures, such as cups, tablespoons, or measurement in grams), and food description (preparation and cooking methods, ingredients of mixed dishes, and specific brand names). To increase accuracy, each participant attended an interview after submitting their 7dFD with the lead researcher in a private room at the team’s training facilities before a training session to review unclear descriptions, errors, or omissions. The food diary records were assessed using Nutritics professional diet analysis software (Nutritics Ltd., Dublin, Ireland). All of the information provided in the food diary was entered into the software solely by the lead researcher to ensure consistency.

### 2.5. Energy Expenditure

Player’s EE was measured using the SenseWear Pro armband (SWA; BodyMedia, Pittsburgh, PA, USA). Athletes wore the armband 24 h a day for seven days (the same consecutive seven days for which players recorded their dietary intake), except during bathing. The armband was worn on the back of the upper right arm. The SWA is composed of multiple sensors that include a two-axis accelerometer, heat flux sensor, galvanic skin response sensor, skin temperature sensor, and a near-body ambient temperature sensor. The SWA proprietary software (Sensewear Professional 8.0; BodyMedia) was used to calculate energy expenditure based on a proprietary algorithm that incorporates the subject information entered before data collection (height, weight, age, and gender) and measurements collected by the armband. SWA has been demonstrated to be a valid device for estimating EE in free-living individuals and during physical activity (up to 10 metabolic equivalents) [24,25]. Brazeau et al. [26] evaluated the reliability of the SWA, and found the device to be reliable for estimating EE in healthy adults during two consecutive days while they were performing various types of activities, including a 45-min walking session and a 45-min cycling session at moderate intensity. Results from this study revealed a coefficient of variation percentage (CV%) of <1%.

### 2.6. Identifying Records of Poor Validity: Misreporting

Day-to-day variation in EI and in EE is normal, and the exact agreement between EI and EE over seven days in one individual is unlikely. Therefore, the accuracy of the reported EI was assessed using the confidence limits (CLs) of agreement between reported EI and EE at the individual level as suggested by Black [27]. Participants can be classified as under-reporters (UR), acceptable reporters (AR), or over-reporters (OR), according to their individual ratio of reported EI to EE. The 95% CLs of agreement between EI/EE were calculated as:

95% CL = ± 2 √ [(CV^2^_EI_/d) + (CV^2^_EE_)] (1)

where CV_EI_ is the coefficient of variation for the reported EI (23), CV_EE_ is the coefficient of variation for the EE (8.2), and d is the number of days (7). Acceptable reporters were defined as having a ratio EI/EE in the range 76–124%.

### 2.7. Statistical Analysis

All of the data are presented as the mean ± SD. Before analysis, normality was assessed using the Shapiro–Wilk test. Paired samples *t*-test was used to analyse differences in mean seven-day energy balance and differences in energy balance for different types of days (pitch, gym, rest). Daily energy and macronutrient intake were analysed using one-way repeated-measure ANOVAs. When there was a significant (*p* < 0.05) effect of “day”, Bonferroni post hoc pairwise comparisons were performed to identify which day differed. The statistical analysis was carried out using SPSS (Version 24; SPSS Inc., Chicago, IL, USA).

## 3. Results

### 3.1. Participant Characteristics

Participant characteristics are presented in Table 2. Eight athletes were identified as URs, 10 athletes were classified as ARs, while none were categorized as ORs. The eight participants from this study that were identified as under-reporting were excluded from the final dietary analysis. The URs were significantly younger than the ARs (*p* = 0.031). No significant differences for weight (*p* = 0.422) were found between the UR and AR groups. A significant difference in mean weekly EE was shown between the AR and UR groups (*p* = 0.032), with the UR group reporting a greater mean weekly EE.

### 3.2. Energy Intake and Energy Expenditure

**Figure 1:**
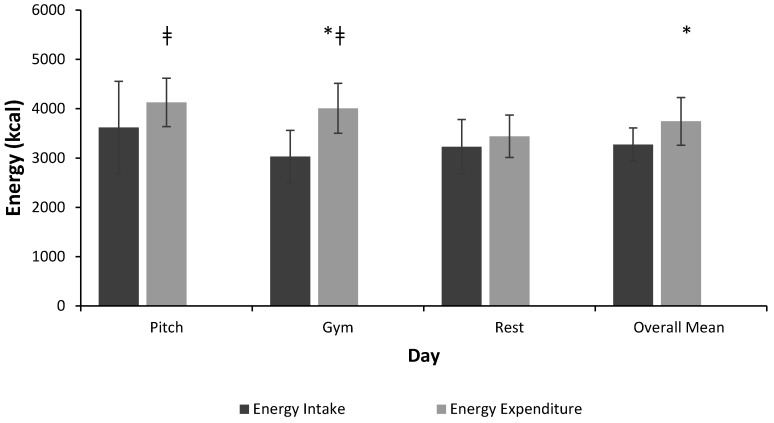
Mean energy intake (kcal) compared to mean energy expenditure (kcal) of acceptable reporters (ARs) for type of day. *: Significant difference between mean energy intake and mean energy expenditure at the corresponding time-point, *p* < 0.05. ǂ: Energy expenditure significantly different from rest day *p* < 0.05 (n = 10).

Mean daily total EE for all players was 3966 ± 506 kcal. Mean total EE for pitch-based training days, gym-based train trainings, and rest days were 4255 ± 515 kcal, 4130 ± 636 kcal, and 3639 ± 480 kcal, respectively. EE was significantly greater on pitch (*p* < 0.001) and gym training days (*p* = 0.05) than rest days. Figure 1 illustrates the mean daily EI and expenditure data of AR based on the type of training day. The mean daily EI (3283 ± 483 kcal) was significantly lower than the mean daily EE (3743 ± 335 kcal) (*p* = 0.016). This resulted in a mean daily energy deficit of −460 ± 503 kcal. A significant difference was observed between mean EI and EE on gym training days (*p* = 0.02). Although an energy deficit was also observed on pitch training days (*p* = 0.059) and rest days (*p* = 0.3), this was not statistically significant. No statistically significant differences were identified in EI with respect to the type of day throughout the week.

### 3.3. Macronutrient Profile

**Figure 2:**
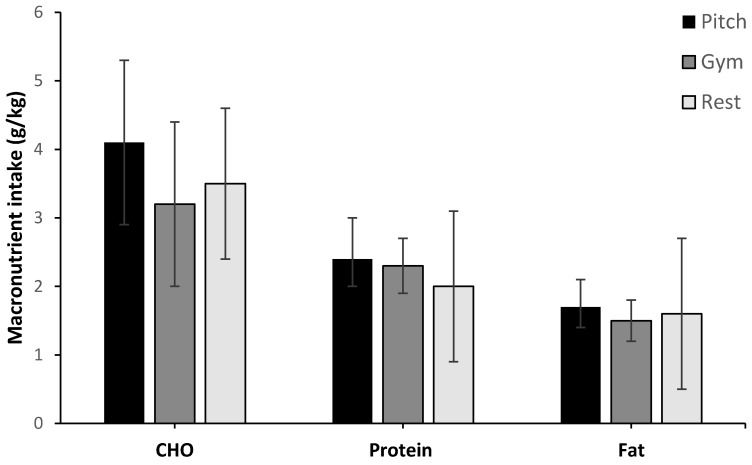
Daily macronutrient intake expressed relative to body mass for type of training day (n = 10). No difference between days.

Total and relative macronutrient intake compared with sport nutrition recommendations are shown in Table 3. The mean daily macronutrient intakes, with a breakdown in relation to type of training day, are expressed in Figure 2. No significant difference was observed for macronutrients between days.

## 4. Discussion

This is the first study to use wearable technology to assess EE alongside quantifying EI in elite Gaelic footballers. As hypothesized, the findings indicate that Gaelic football players were in negative energy balance, consumed less CHO than recommended, and failed to adjust dietary intake relative to needs during the pre-season training week. Despite the authors’ best efforts to minimise the under-reporting of dietary intake, the discrepancy between EE and EI, alongside players’ body mass remaining relatively unchanged during the study, indicates that under-reporting may have occurred. The total EI was similar to that reported in previous published research studies that have assessed dietary intake within Gaelic footballers [14,15]; however, these studies did not measure EE. In the present study, the type of day had an influence on the magnitude of energy deficit. Training days were a particular risk to energy balance, with players not adjusting EI to encounter the increased energy cost of training. This data could be of use to practitioners and players who should consider adjusting EI accordingly.

The mean daily EE reported in this study is higher when compared to mean daily EE reported for elite rugby union [19] and soccer players [20]. However, the EE reported in this study was lower than that reported for a group of professional rugby league players [28]; therefore, nutritional guidelines for team sports should be tailored to the specific sport and athlete. Despite elite Gaelic football players being amateur, they train at a similar level to professional athletes [9]. Several players who were assessed during this study had active day jobs; this alongside the heavy training demands will result in high levels of EE. Sports nutritionists working with elite Gaelic footballers should acknowledge the demands of their professional lives (work/study) alongside training demands and adjust their dietary advice accordingly.

As expected, EE varied during the week, with higher EE elicited during training days compared to rest days. The present findings suggest that EI and macronutrient consumption is relatively stable across the week; therefore, players are not adjusting intake to account for the intensity of training. This finding supports previous research, albeit in the professional rugby league, whereby players’ EI was not adjusted for type of training day; in addition, energy derived from CHO remained consistent throughout the week [16]. Periodised dietary intake should be recommended to account for both training intensity and volume; players should adjust their energy and macronutrient intake according to the type of training day in order to increase performance and maintain health [13]. The periodisation of training is a recognised concept in sports science; however, dietary periodisation is a developing concept in sports nutrition, which acknowledges that an athlete’s nutritional intake should vary according to their training and competition demands [13].

The average CHO intake observed in the present study was 3.6 ± 0.7 g/kg/day. This corresponds with previous research that reported the dietary intakes of Gaelic footballers [14,15]. The mean dietary CHO intake fell below the lower range of sports nutrition guidelines (5–10 g/kg/day) [13]. The CHO intake remained consistent during the week, and was not altered to account for training load. High-CHO diets result in increased muscle glycogen stores, which consequently delays the onset of fatigue and sustains performance levels during high-intensity exercise [29]. Chronic low CHO availability can result in reduced immune function, increased muscle breakdown, and a reduced capacity to use CHO as fuel [30]. As CHO intake is crucial for performance, nutritional education strategies for elite Gaelic footballers should focus on helping players achieve adequate intakes of CHO.

By using a similar approach to Brinkman et al. [31], the measurement of EE in the present study can allow us to evaluate CHO intake recommendations for Gaelic footballers in relation to daily EE. The mean EE of players in the current study for pitch-based training days, gym-based train trainings, and rest days were 4255 ± 515 kcal, 4130 ± 636 kcal, and 3639 ± 480 kcal, respectively. CHO intakes of 5–7 g/kg/day are recommended for moderate intensity training (~1 h per day) [13]. The upper limit of CHO intake guidelines for moderate intensity training (up to 7 g/kg/day) may be appropriate to match the increased energy requirement of pitch training days during the pre-season. When considering a Gaelic footballer of 84 kg (the mean body mass of the current study), a daily CHO intake of 7 g/kg body mass translates to 2352 kcal. When accounting for the current study’s findings of mean protein intake of 2.1 g/kg/day (706 kcal), and fat intake of 1.6 g/kg/day (1210 kcal), the total daily EI would equate to 4268 kcal, which would result in players achieving energy balance. To achieve energy balance on gym and rest days, players should consume CHO intakes of 6.5 g/kg/day and 5 g/kg/day, respectively. Our findings suggests that current recommendations to consume up to 7 g/kg body mass/day of CHO may be sufficient to fuel training and achieve energy balance during the pre-season training period. We feel that our modified CHO recommendations are more specific, realistic, and achievable for Gaelic football players than existing recommendations.

Protein is an essential macronutrient for recovery and to support increases in lean muscle mass [22]. The current study’s findings of 2.1 ± 0.5 g/kg/day is greater than the guidelines of 1.2–2.0 g/kg/day for athletes [13]; however, this is similar to protein intakes reported in professional rugby union players [32]. To preserve muscle mass whilst decreasing body fat, protein intakes of 2.3 g/kg/day have been advised [33]. One of the main objectives of elite Gaelic footballers during the pre-season period is to maintain or increase lean muscle mass. There are no reported risks of ingesting these high intakes of protein in a non-diseased state [34]; therefore, it could be suggested that the reported protein intakes of the players in this study were suitable.

The mean daily dietary fat intake in the present study (37.5 ± 3.6%) is marginally greater than that of recommended consumption [13], which could explain the inadequate intake of optimal levels of carbohydrate. Higher proportions of energy derived from fat and protein sources can compromise the total intake of CHO [35]. Players should focus on increasing the consumption of CHOs, as this would have a simultaneous effect of decreasing the amount of energy resulting from total fat intake.

Although this study provides novel and valuable data, there are some limitations. Under-reporting is an inherent limitation of dietary assessment in athletes [36]. There is no gold standard measurement of EI, any method used is subject to error; hence, dietary intake data must be treated with caution [37]. Despite the authors’ best efforts to minimise the under-reporting of dietary intake, there was still a significant negative energy balance. Self-reported EI bias can be as great as 36% in athletes [28,38,39]. Drenowatz et al. [40] suggested that the increased energy demands of athletes and their increased number of eating occasions can contribute to under-reporting. The SWA has been shown to accurately estimate EE at rest, low, and moderate intensity exercise [25,41]; however, at higher intensities, the SWA has been shown to underestimate EE [26,42], and this should be considered when interpreting the data. Another limitation was that the data was only collected from a small sample of Gaelic football players on one elite team, and therefore, the data may not represent every elite Gaelic football squad. Future studies should collect data from a variety of teams.

## 5. Conclusions

The present study has for the first time attempted to assess the dietary intake and EE of elite Gaelic footballers during a typical pre-season week. The nutritional practices of the sampled players were inadequate to meet energy demands and CHO recommendations. Specifically, training days are of concern, with players not altering EI and CHO to match the increased energy demands of training. Nutrition goals and requirements are not static; different days have different demands, and therefore different nutritional requirements. Gaelic football teams undertake periodised training programs where players perform different types of workouts across different phases of training in preparation for peak performance. Nutritional intake also needs to be periodised, considering the daily needs of the training session.

## Figures and Tables

**Table 1:** Typical pre-season training week.

Monday	Tuesday	Wednesday	Thursday	Friday	Saturday	Sunday
Resistance Training (Gym) (60 min)	Off	Resistance Training (Gym) (60 min)	Off	Pitch Conditioning and Football (90 min)	Off	Pitch Conditioning and Football (90 min)

**Table 2:** Participant characteristics and average daily energy expenditure (EE) during the study period.

Parameters	Total (n = 18) Mean ± SD	AR (n = 10) Mean ± SD	UR (n = 8) Mean ± SD
Age (years)	23.7 ± 3.1	25.0 ± 2.9 *	22.1 ± 2.6
Weight (kg)	84.6 ± 8.0	86.1 ± 7.3	82.9 ± 9.1
Height (cm)	183.3 ± 6.2	183.8 ± 6.5	182.6 ± 6
Average daily EE (kcal)	3966 ± 506	3743 ± 335 *	4245 ± 563

* Significantly different than UR.

**Table 3:** Mean daily macronutrient intakes compared with sport nutrition recommendations (n = 10).

Macronutrient		Intake (Mean ± SD)	Sport Nutrition Recommendation
Carbohydrate	Total (g)	305.7 ± 57.1	-
	g/kg body mass	3.6 ± 0.7	5–10 g/kg/d [13]
	% total energy intake	37.7 ± 4.5	-
Protein	Total (g)	184.9 ± 40.2	-
	g/kg body mass	2.1 ± 0.5	1.2–2.0 g/kg/d [13]
	% total energy intake	23.2 ± 4.0	-
Fat	Total (g)	135.8 ± 23.9	-
	g/kg body mass	1.6 ± 0.2	-
	% total energy intake	37.5 ± 3.6	20–35% total energy [13]
